# The Impact of Caffeine and Coffee on Human Health

**DOI:** 10.3390/nu11020416

**Published:** 2019-02-16

**Authors:** Marilyn C. Cornelis

**Affiliations:** Department of Preventive Medicine, Northwestern University Feinberg School of Medicine, Chicago, IL 60611, USA; Marilyn.cornelis@northwestern.edu; Tel.: +1-312-503-4548

Coffee is one of the most widely consumed beverages in the world and is also a major source of caffeine for most populations [1]. This special issue of *Nutrients*, “The Impact of Caffeine and Coffee on Human Health” contains nine reviews and 10 original publications of timely human research investigating coffee and caffeine habits and the impact of coffee and caffeine intake on various diseases, conditions, and performance traits.

With increasing interest in the role of coffee in health, general knowledge of population consumption patterns and within the context of the full diet is important for both research and public health. Reyes and Cornelis [1] used 2017 country-level volume sales (proxy for consumption) of caffeine-containing beverages (CCBs) to demonstrate that coffee and tea remain the leading CCBs consumed around the world. In a large coordinated effort spanning 10 European countries, Landais et al. [2] quantified self-reported coffee and tea intakes and assessed their contribution to the intakes of selected nutrients in adults where variation in consumption was mostly driven by geographical region. Overall, coffee and tea contributed to less than 10% of the energy intake. However, the greatest contribution to total sugar intake was observed in Southern Europe (up to ~20%). These works not only emphasize the wide prevalence of coffee and tea drinking, but also the need for data on coffee and tea additives in epidemiological studies of these beverages in certain countries as they may offset any potential benefits these beverages have on health.

Doepker et al. [3] provided a user-friendly synopsis of their systematic review [4] of caffeine safety, which concluded that caffeine doses (400 mg/day for healthy adults, for example) previously determined in 2003 [5] as not to be associated with adverse effects, remained generally appropriate despite new research conducted since then. Further concerning caffeine safety is the systematic review of caffeine-related deaths by Capelletti et al. [6]. Suicide, accidental, and intentional poisoning were the most common causes of death and most cases involved infants, psychiatric patients, and athletes. Both Doepker et al. [3] and Capelletti et al. [6] alluded to the increasing interest in the area of between-person sensitivity resulting from environmental and genetic factors, of which the latter is a topic of additional papers in this special issue and thus reiterates this interest.

Advancements in high-throughput analyses of the human genome, transcriptome, proteome, and metabolome have presented coffee researchers with an unprecedented opportunity to optimize their research approach while acquiring mechanistic and causal insight to their observed associations [7]. Three timely reviews [8,9,10] and an original report [11] addressed the topic of human genetics and coffee and caffeine consumption. Interest in this area received a boost by the success of genome-wide association studies (GWAS), which identified multiple genetic variants associated with habitual coffee and caffeine consumption as discussed by Cornelis and Munafo [8] in their review of Mendelian randomization (MR) studies on coffee and caffeine consumption. MR is a technique that uses genetic variants as instrumental variables to assess whether an observational association between a risk factor (i.e., coffee) and an outcome aligns with a causal effect. The application of this approach to coffee and health is growing, but has important statistical and conceptual challenges that warrant consideration in the interpretation of the results. Southward et al. [9] and Fulton et al. [10] reviewed the impact of genetics on physiological responses to caffeine. Both emphasized a current clinical interest limited to *CYP1A2* and *ADORA2A* variations, suggesting opportunities to expand this research to more recent loci identified by GWAS. Despite the advancements in integrating genetics into clinical trials of caffeine, such designs remain susceptible to limitations [9,10,12,13]. Some of these limitations were further highlighted by Shabir et al. [14] in their critical review on the impact of caffeine expectancies on sport, exercise, and cognitive performance. Interestingly, the original findings from a randomized controlled trial of regular coffee, decaffeinated coffee, and placebo suggested the stimulant activity of coffee beyond its caffeine content, raising issues with the use of decaffeinated coffee as a placebo [15].

The impact of coffee intake on gene expression and the lipidome were investigated by Barnung et al. [16] and Kuang et al. [17], respectively. Barnung et al. [16] reported on the results from a population-based whole-blood gene expression analysis of coffee consumption that pointed to metabolic, immune, and inflammation pathways. Using samples from a controlled trial of coffee intake, Kuang et al. [17] reported that coffee intake led to lower levels of specific lysophosphatidylcholines. These two reports provide both novel and confirmatory insight into mechanisms by which coffee might be impacting health and further demonstrate the power of high-throughput omic technologies in the nutrition field.

Heavy coffee and caffeine intake continue to be seen as potentially harmful on pregnancy outcomes [18]. Leviton [19] discussed the biases inherent in studies of coffee consumption during pregnancy and argued that all of the reports of detrimental effects of coffee could be explained by one or more of these biases. The impact of dietary caffeine intake on assisted reproduction technique (ART) outcomes has also garnered interest. An original report by Ricci et al. [20] in this special issue found no relationship between the caffeine intake of subfertile couples and negative ART outcomes.

Van Dijk et al. [21] reviewed the effects of caffeine on myocardial blood flow, which support a significant and clinically relevant influence of recent caffeine intake on cardiac perfusion measurements during adenosine and dipyridamole induced hyperemia. Original observational reports on the association between habitual coffee consumption and liver fibrosis [22], depression [23], hearing [24], and cognition indices [25] have extended the research in these areas to new populations.

Finally, given the widespread availability of caffeine in the diet and the increasing public and scientific interest in the potential health consequences of habitual caffeine intake, Reyes and Cornelis [1] assessed how current caffeine knowledge and concern has been translated into food-based dietary guidelines (FBDG) from around the world; focusing on CCBs. Several themes emerged, but in general, FBDG provided an unfavorable view of CCBs, which was rarely balanced with recent data supporting the potential benefits of specific beverage types.

This collection of original and review papers provides a useful summary of the progress on the topic of caffeine, coffee, and human health. It also points to the research needs and limitations of the study design, which should be considered going forward and when critically evaluating the research findings.
